# Bioinspired Spatially Ordered Multicellular Lobules for Liver Regeneration

**DOI:** 10.34133/research.0634

**Published:** 2025-03-17

**Authors:** Jinglin Wang, Danqing Huang, Haozhen Ren, Yuanjin Zhao

**Affiliations:** ^1^ Division of Hepatobiliary and Transplantation Surgery, Department of General Surgery, Nanjing Drum Tower Hospital, Affiliated Hospital of Medical School, Nanjing University, Nanjing 210008, China.; ^2^ Department of Rheumatology and Immunology, Nanjing Drum Tower Hospital, School of Biological Science and Medical Engineering, Southeast University, Nanjing 210096, China.; ^3^ Institute of Organoids on Chips Translational Research, Henan Academy of Sciences, Zhengzhou 450009, China.

## Abstract

Cell therapy is a promising strategy for acute liver failure (ALF), while its therapeutic efficacy is often limited by cell loss and poor arrangement. Here, inspired by liver microunits, we propose a novel spatially ordered multicellular lobules for the ALF treatment by using a microfluidic continuous spinning technology. The microfluidics with multiple microchannels was constructed by assembling parallel capillaries. Sodium alginate (Alg) solution encapsulating human umbilical vein endothelial cells (HUVECs), hepatocytes, and mesenchymal stem cells (MSCs) are introduced into the middle channel and the 6 parallel outer channels of the microfluidics, respectively. Simultaneously, Ca^2+^-loaded solutions are pumped through the innermost and outermost channels, forming a hollow microfiber with hepatocytes and MSCs alternately surrounding the HUVECs. These microfibers could highly resemble the cord-like structure of liver lobules, bringing about outstanding liver-like functions. We have demonstrated that in ALF rats, our biomimetic lobules can effectively suppress excessive inflammatory responses, decrease cell necrosis, and promote regenerative pathways, leading to satisfied therapeutic efficacy. These findings underscore the potential of spatially ordered multicellular microfibers in treating related diseases and improving traditional clinical methods.

## Introduction

Liver diseases often lead to liver dysfunction and fatal outcomes. Among them, acute liver failure (ALF) is a serious condition characterized by widespread hepatocyte necrosis [1–3]. Various strategies have been employed to release severe injury in ALF, with cell transplantation therapy being considered the most promising approach [4–6]. This therapy involves the application of external cells, such as hepatocytes and mesenchymal stem cells (MSCs), to promote liver repair [7–10]. Generally, the transplanted hepatocytes can partially restore liver functions, including protein synthesis, glycogen storage, and detoxification, facilitating rapid recovery [11–13]. In comparison, MSCs have paracrine functions, secreting cytokines that suppress inflammation, regulate immune responses, and accelerate repair at the site of injury [14]. However, conventional intravenous infusion of these cells often results in severe cell loss before reaching the damaged area [15,16]. Although the use of hydrogel encapsulation could achieve in situ transplantation, fully exploiting these cells’ functionality remains challenging [17,18]. In addition, due to the complex structure of the liver, relying solely on one type of cell transplantation is heavily influenced by the internal environment of the recipient and often fails to achieve the desired therapeutic effect [19]. Therefore, the development of new cell delivery strategies is necessary to improve the survival and functionality of cell transplantation for ALF.

**Fig. 1.**
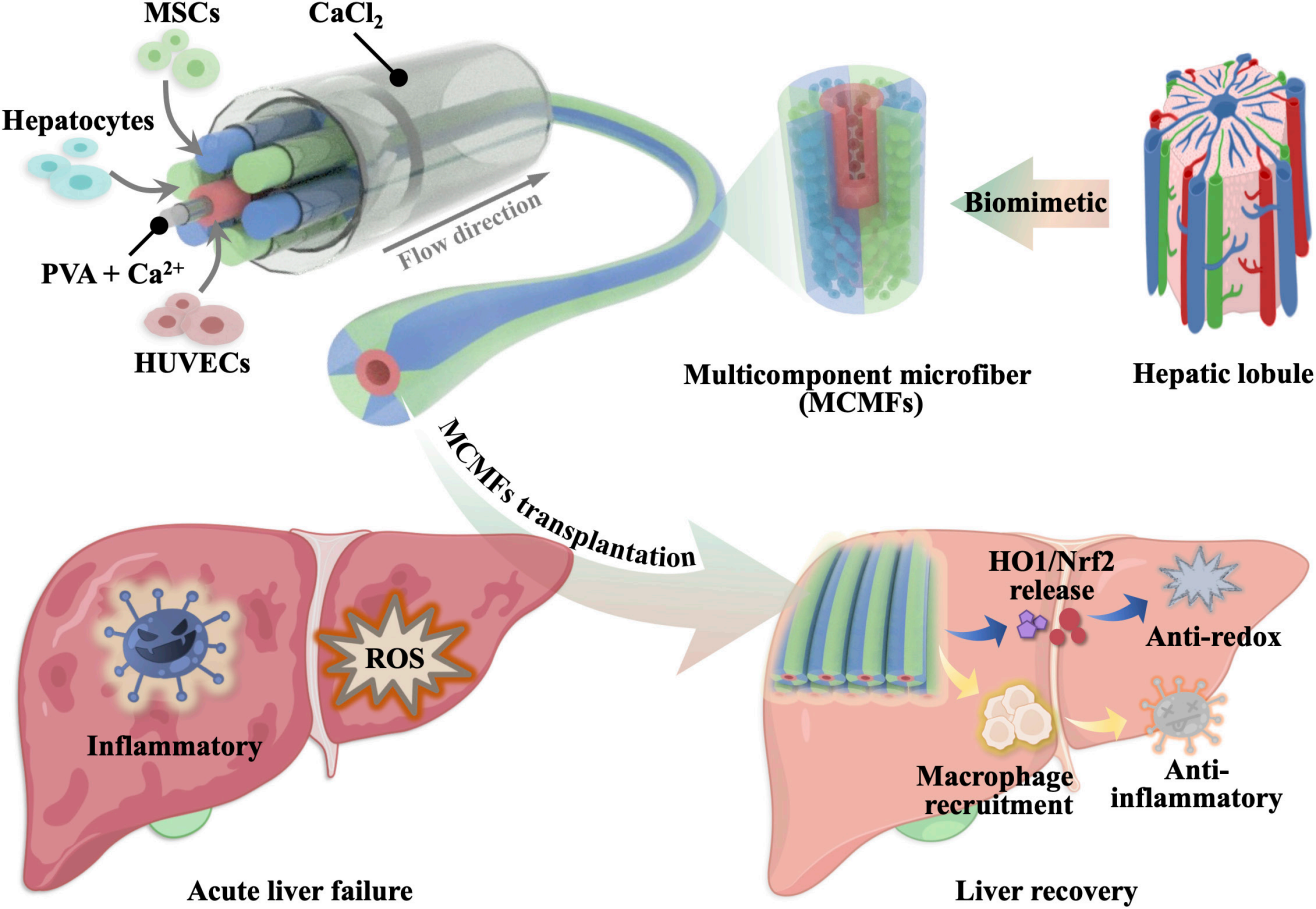
Schematic illustration of the continuously produced MCMFs by microfluidic technology. The MCMFs can modulate the hyperactivated ROS and reprogram macrophages to suppress the inflammatory storm during treatment of ALF.

Here, inspired by the natural structure of liver microunits, we propose a novel spatially ordered multicellular lobules through continuous microfluidic spinning for the transplantation therapy of ALF, as shown in Fig. 1. Generally, hepatic parenchymal and non-parenchymal cells arrange radially around the central vein in single liver lobule [20,21]. Ascribing to their hollow tubular channels, these liver lobules provide a spacious distribution area and a stable framework for various colonized cells [22]. Additionally, their axial channels can also effectively deliver oxygen and nutrients to cells, promoting cell metabolism and growth. To fully realize the therapeutic efficacy of cell transplantation, numerous studies have focused on constructing microcarriers that mimic the structure of liver lobules to encapsulate cells [23]. Despite advanced progress, continuous generation of the biomimetic liver lobules remains unachievable, limiting the mass production of complex structures [24,25]. In addition, recent research has merely imitated each other, making geometric modifications without truly simulating the spatiotemporal distribution of liver lobule cells [26,27]. Thus, it remains challenging to develop an effective approach to construct highly realistic lobule structures and expand their applications.

In this study, we employed a multichannel microfluidic spinning platform to fabricate the spatially ordered multicellular lobules for ALF treatment. To mimic the lobular structure of the liver, we designed a glass-based coaxially assembled microfluidic chip with the same cross-sectional structure. Ascribing to the rapid cross-linking of Ca^2+^ and alginate (Alg) solution, we can instantly obtain cell-encapsulated Ca-Alg microfibers. By introducing different cell-loaded Alg solutions into different channels, we achieved the organized spatial assembly of multiple cell types within the microfibers. In particular, by alternately pumping hepatocyte- and MSC-loaded Alg solutions into 6 parallel outer channels, human umbilical vein endothelial cell (HUVEC)-loaded Alg solution into the middle channel, we obtained multicellular microfibers (MCMFs) with a hollow structure. In these MCMFs, hepatocytes and MSCs alternately surrounded HUVECs, resembling the lobular structure of the liver. Based on this, we performed a transplantation of these MCMFs for ALF treatment to assess their functionality. Our MCMFs were demonstrated to create an optimized microenvironment that can minimize cell necrosis during treatment and facilitate regenerative pathways for liver recovery. Moreover, these MCMFs exhibited the ability to reduce oxidative stress and suppress excessive inflammatory responses, markedly elevating therapeutic efficacy. Thus, the proposed MCMFs showed practical value in liver disease treatment and provided an effective way for constructing biomimetic cell carriers.

## Results and Discussion

**Fig. 2.**
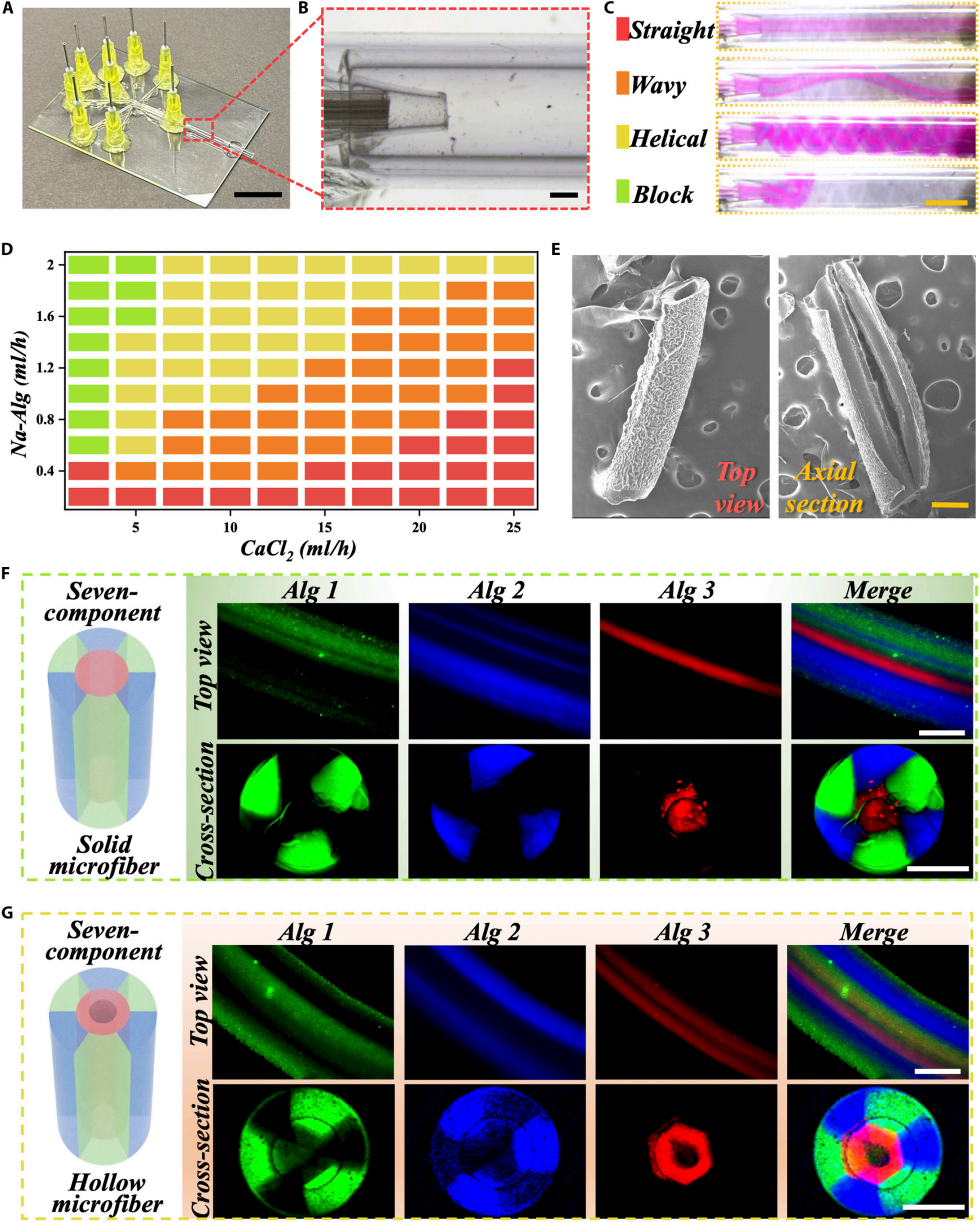
Establishment of microfluidic chip and fabrication of microfibers. (A) Photograph of the coflow microfluidic chip platform by spatially assemble capillary glass tubes. Scale bar, 1 cm. (B) Stereomicroscopic images of the orifice in the assembly of parallel capillary glasses (Tube IV in Tube V). Scale bar, 1 mm. (C) The microfibers formed by microfluidics in different velocities of the Na-Alg and outer CaCl_2_ solution can be classified into 4 main states. Scale bar, 2 mm. (D) The flow velocities of the Na-Alg in Tubes I and II and CaCl_2_ solution in Tube V regulate the microfiber states. (E) Scanning electron microscope (SEM) images of overall morphology (left) and the cross-sectional view along the longitudinal axis (right) of hollow microfibers. Scale bar, 500 μm. (F and G) Laser scanning confocal microscope (LSCM) images of the top view and cross-sectional view of the solid (F) and hollow (G) microfibers. The Ca-Alg fluorescence is labeled as Alg1 (green), Alg2 (blue), and Alg3 (red). Scale bars are 500 μm.

In a typical experiment, we fabricated a coflow microfluidic chip platform by spatially assembling capillary glass tubes with different diameters (Fig. 2A). Firstly, we assembled 7 capillary glass tubes with an inner diameter of 500 μm parallelly in a 2-3-2 arrangement from top to bottom, as shown in Fig. S1A. The outer ring of 6 tubes is collectively referred to as Tube I, while the single capillary glass tube in the middle is referred to as Tube II. In the middle of Tube II, we inserted another capillary glass tube with an inner diameter of 120 μm (referred to as Tube III), forming a circular array of capillary glass tubes. These combined Tubes I to III were inserted into a capillary glass tube with an inner diameter of 2.4 mm, the outlet of which was tapered into a diameter of 1 mm (referred to as Tube IV) (Fig. S2). Subsequently, we constructed the microfluidic chip layer by layer, starting from the outermost layer. The round tube (referred to as Tube V) was fixed in an appropriate position on the glass slide, leaving sufficient space for the other tubes on the slide (Fig. S1B). Then, combined Tubes I to IV and a capillary tube with an inner diameter of 1.5 mm (referred to as Tube VI) were inserted in reverse through Tube V, ensuring that the tip of Tube IV inserted into Tube V without completely blocking its opening, and the 2 tubes were secured (Fig. 2B). To introduce different fluids and achieve diversity in microfiber components, 6 tapered and curved capillaries were inserted into Tube I individually (Fig. S3A). Finally, needles were fixed at the junctions of all the curved tubes to facilitate fluid introduction.

Since the rapid gelation reaction between sodium alginate (Na-Alg) and Ca^2+^, they are commonly employed to fabricate complex calcium alginate (Ca-Alg) microfiber structures through microfluidic techniques. To obtain the desired microfibers, a Na-Alg solution was introduced into the chip device through the middle channel (Tube I, gaps between Tubes II and III), while calcium chloride (CaCl_2_) fluid is introduced into the device through the outer channels (gaps between Tubes IV and VI). This allowed for in situ cross-linking, resulting in the formation of solid multicomponent microfibers. Due to the device’s multiple arrays of capillary glass tubes, various combinations of microfiber compositions can be obtained. To validate the diversity of microfiber compositions, we alternately pumped Na-Alg solution stained with green and blue fluorescence into the 6 channels of Tube I, while CaCl_2_ flowed into the gaps between Tubes IV and VI. By sealing the other channels, we were able to obtain solid microfibers with a cross-section composed of 6 fan-shaped structures, featuring alternating green and blue fluorescence, and each with distinct boundaries (Fig. S4). Building upon this, we further enhanced the spatial composition of the solid microfibers. By opening the channel between Tubes II and III and introducing Na-Alg solution stained with red fluorescence, we achieved solid microfibers with a total of 7 components. In the cross-section of these microfibers, a central microfiber with red fluorescence will be surrounded by a ring of microfibers alternating between green and blue fluorescence (Fig. 2F and Fig. S5).

To mimic the hollow vertical structure of hepatic lobules, we introduced Ca^2+^-loaded polyvinyl alcohol (PVA) solution as the inner phase (Tube II) in the abovementioned microfluidic system (Fig. S6). PVA solution is an inert water-soluble solution that does not react with Na-Alg or CaCl_2_ solution. The fluid occupied by the PVA solution forms the inner channel. Ascribing to PVA’s viscosity, which is comparable to that of Na-Alg solution, diffusion between the 2 phases is effectively reduced, ensuring the formation of the innermost channel. Besides, PVA exhibits excellent biocompatibility and does not cause harm to cells in subsequent cell experiments. During the experimental process, we observed that the microfibers formed within the microfluidic channels are not always straight. By adjusting the fluid velocities of Na-Alg and outer CaCl_2_ solutions, the microfibers exhibit 4 main states: straight, wavy, helical, and blocked (Fig. 2C and D). As the velocity of the Na-Alg phase increased and the velocity of the CaCl_2_ phase decreased to a certain ratio, the straight microfiber state disappeared, and randomly helical flow patterns gradually appeared. Among these 4 states, straight microfibers are the most stable state and have a stability range that increases in length and shifts to the right as the velocity of CaCl_2_ phase increases. After fine-tuning the velocities of the PVA, Na-Alg, and outer CaCl_2_ solutions, we successfully obtained stable, straight, and hollow microfibers, as depicted in Fig. 2E and Fig. S3B. From the fluorescence images, it was evident that these microfibers have a hollow structure with a double-layered tubular configuration (Fig. 2G). Notably, the outer layer of the microfibers met the requirements for diverse spatial compositions. This achievement of generating hollow microfibers with a dual-layered tubular structure opens up exciting possibilities for incorporating a wide range of spatial components.

**Fig. 3.**
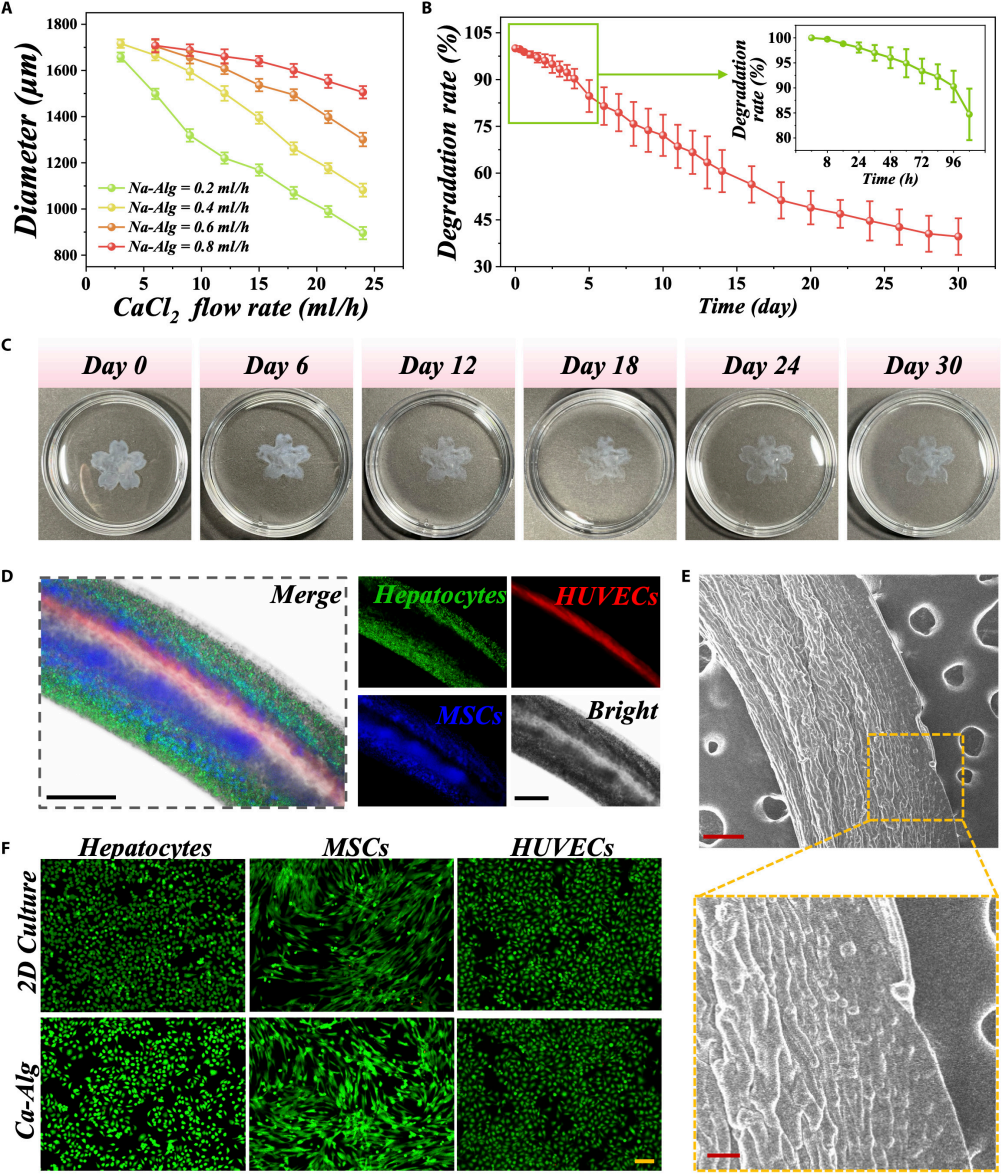
Cell distribution and growth of MCMFs. (A) The statistical graph illustrating the relationship between the flow rate of Na-Alg and outer CaCl_2_ phases and the diameter of the microfibers. (B and C) In vitro degradation curve (B) and actual images (C) of Ca-Alg. (D) Fluorescence images of MCMFs. Scale bars are 500 μm. (E) SEM images of MCMFs are provided, with a scale bar of 200 μm for the upper image and 50 μm for the lower magnified region. (F) The merged live-dead staining images of hepatocytes, MSCs, and HUVECs in 2D culture and the Ca-Alg group. Green fluorescence represents live cells, while red fluorescence represents dead cells. Scale bar, 100 μm.

Encouraged by the above results, we could achieve stable microfiber generation by adjusting the flow rates of the Na-Alg and outer CaCl_2_ phases. In fact, the flow rate not only determines the microfiber generation conditions but also affects the microfiber sizes. As shown in Fig. 3A, the microfiber size decreased with increasing CaCl_2_ flow rate. For example, when the Na-Alg flow rate through each capillary channel was fixed at 0.2 ml/h, the microfiber size decreases from 1,600 to 900 μm approximately. Conversely, the microfiber size decreased with increasing Na-Alg flow rate. Therefore, under fixed experimental conditions, the range of microfiber size adjustment was limited. By modifying the experimental parameters, we could achieve microfiber size adjustment within different ranges. Since the Ca-Alg hydrogel is a cross-linked polymer material composed of a 3-dimensional (3D) network structure and the aqueous phase medium within its network pores, it exhibits good biodegradability. Based on this, we evaluated the in vitro degradation of the Ca-Alg hydrogel. With the passage of time, a gradual reduction in the mass of the hydrogel was observed (Fig. 3B and C). After the first 4 days, the hydrogel maintained 90% of its initial mass, and by the 14th day, it had degraded by more than 40%. This indicates that the Ca-Alg hydrogel provides a favorable microenvironment for cell growth throughout the acute disease process.

To enhance the biomimetic functionality of Ca-Alg microfibers, we focused on utilizing the coflow microfluidic chip platform and microfluidic pumps for the spatially ordered assembly of cells. Research has found that the combination of various types of liver cells in vitro can promote hepatocyte functionality. However, current methods only involve mixing cell types and cannot simulate the spatial structure and composition distribution of liver lobules. To achieve a more realistic simulation of the in vivo pattern of liver lobules, we introduced a Na-Alg solution containing hepatocytes (labeled with green fluorescence) and MSCs (labeled with blue fluorescence) into the 6 channels of Tube I at regular intervals. Simultaneously, a Na-Alg solution containing HUVECs (labeled with red fluorescence) was pumped into the gaps between Tube II and Tube III channels. Additionally, by introducing CaCl_2_ into the gap between Tubes IV and VI and using Ca^2+^-loaded PVA solution as the inner phase (Tube II), we achieved continuously in situ generation of MCMFs with a hollow structure. In these MCMFs, hepatocytes and MSCs alternate in surrounding HUVECs, accurately mimicking the spatially ordered distribution of cells in liver lobule (Fig. 3D). This arrangement fully replicates the cord-like architecture of liver cells around hepatic veins, separated by liver sinusoids. To characterize the morphology of MCMFs and the distribution of cells within the fibers, we performed scanning electron microscope (SEM) imaging. We observed that the cells were randomly and uniformly encapsulated within the microfibers (Fig. 3E). The Ca-Alg microfibers have an interconnected network structure, allowing the flow of water, nutrients, and biomolecules such as proteins, enzymes, and growth factors within the hydrogel’s network. This structure resembles the 3D environment in which cells grow in the body. To validate the viability of cells within the Ca-Alg, we conducted live-dead staining on hepatocytes, MSCs, and HUVECs. The results showed that the cell viability within the Ca-Alg was comparable to or even better than cells cultured on conventional plates, indicating that live cells encapsulated within the Ca-Alg maintained good biological activity (Fig. 3F and Fig. S7).

**Fig. 4.**
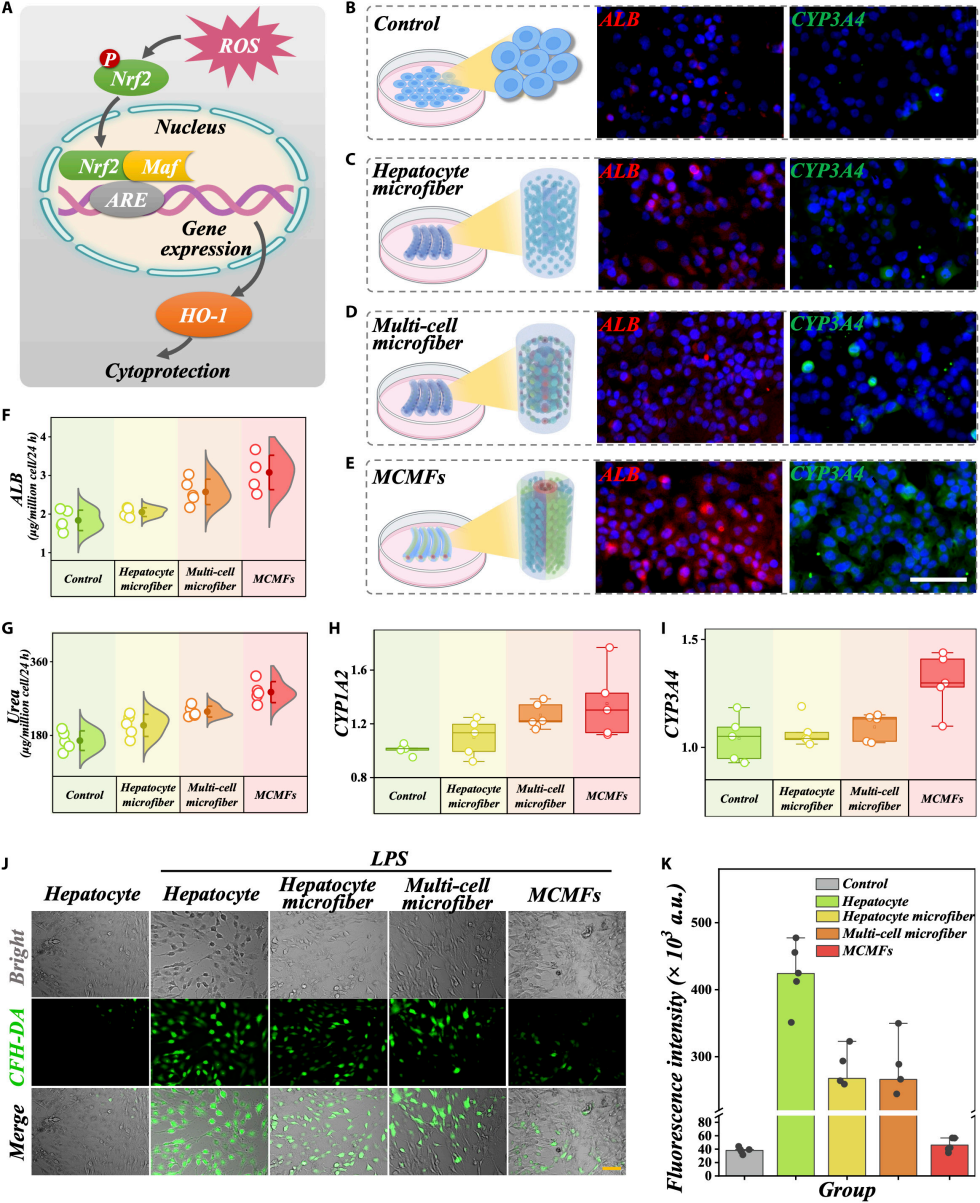
Comparing the in vitro functions of MCMFs, as well as the detection of ROS levels under LPS stimulation. (A) The diagram illustrates the production of ROS during ALF and the protective effect of the Nrf2/HO-1 pathway on cells. (B to E) Immunofluorescence analysis of ALB and CYP expression in the control, hepatocyte microfiber, multicell microfiber group, and MCMF groups. Scale bar, 100 μm. (F and G) ELISA detection of ALB (F) and urea (G) synthesis levels in the 4 groups. (H and I) mRNA levels of CYP in the 4 groups. (J and K) ROS levels and quantification in each group after lipopolysaccharide (LPS)-induced acute cell injury. Scale bar, 100 μm in (J).

To further validate the biological functionality of our designed spatially ordered assembly MCMFs, we compared the functional support of hepatocytes using different cell culture methods, primarily focusing on the expression of albumin (ALB) and CYP3A4. In our study, hepatocytes were randomized into 4 groups: control group; hollow microfibers loading hepatocytes (hepatocyte microfiber group); hollow microfibers loading hepatocytes, MSCs, and HUVECs (multicell microfiber group); and MCMFs. We conducted in vitro culture experiments and observed that the immunofluorescence expression levels of ALB and CYP3A4 were higher in the other 3 groups compared to the control group (Fig. 4B to E and Fig. S8). This suggests that hepatocytes perform better in the microfiber environment. Additionally, compared to the random mixture of multiple cell types, MCMFs exhibited higher expression levels of ALB and the CYP family, suggesting that an ordered spatial arrangement of cells could promote liver cell functionalities. Next, we quantitatively measured the ALB synthesis capacity using enzyme-linked immunosorbent assay (ELISA), which confirmed the results obtained from immunofluorescence (Fig. 4F). ALB secretion was found to be the highest in the MCMF group. Additionally, through quantitative ELISA testing of urea, we discovered that MCMFs possess strong urea synthesis capability, indicating an enhancement in the synthetic biological functions of liver cells (Fig. 4G). Furthermore, mRNA testing of the CYP family demonstrated enhanced liver functionality, including stronger drug metabolism (Fig. 4H and I). These findings strongly indicate the potential of MCMFs as a multicellular therapeutic approach for ALF.

When liver cells are stimulated by endogenous or exogenous factors, they can undergo degeneration and necrosis, leading to liver damage. Oxidative stress is one of the important mechanisms underlying liver injury [28–30]. Normally, low levels of reactive oxygen species (ROS) are not cytotoxic, but when pathological changes occur, oxidative stress damage can worsen disease progression due to sustained increases in ROS. To protect the body’s tissues and cells from oxidative stress damage, the body can activate the transcription factor Nrf2, which regulates the antioxidant defense system, to induce increased expression of Nrf2 and its downstream gene HO-1, thereby suppressing liver inflammation and protecting liver cells (Fig. 4A). Recent studies have shown that MSCs have the ability to prevent ALF by clearing ROS [31,32]. Considering this, we simulated ALF in 4 different cell models using LPS induction and studied the potential of MCMFs to clear ROS at the cellular level. We used a fluorescent probe called 7′-dichlorodihydrofluorescein diacetate (DCFH-DA) to detect intracellular ROS. The results demonstrated that compared to liver cells cultured in 2D, the microfiber structure effectively reduced cellular ROS (Fig. 4J and K). Moreover, in the presence of MSCs in the multicell microfiber group and the MCMF group, intracellular ROS levels were markedly reduced, indicating the strong antioxidant activity of MSCs. Importantly, MCMFs showed remarkable ROS-clearing ability, suggesting that the spatially ordered structure importantly contributes to their antioxidant function.

**Fig. 5.**
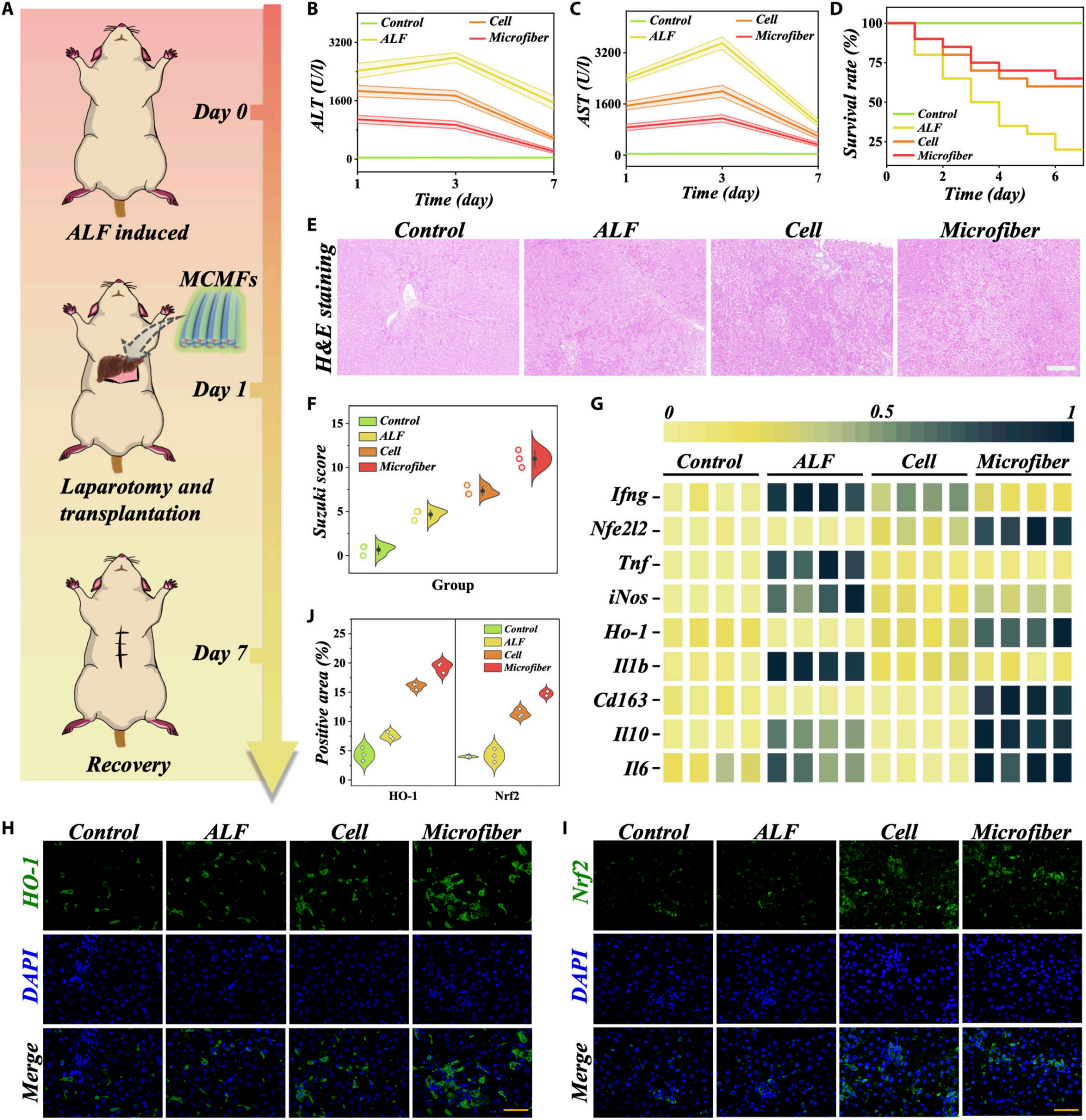
MCMFs transplantation therapy alleviates inflammatory response and oxidative stress in rats with ALF. (A) The schematic illustrates the process of MCMFs transplantation in ALF rats. (B to D) The statistical graphs depict the levels of ALT (B), AST (C), and survival rate (D) in the 4 groups of rats over a period of 7 days. (E and F) The H&E staining images of the liver (E) and corresponding Suzuki scores (a scoring system to assess liver necrosis) (F) are also presented. Scale bar, 200 μm in (E). (G) qRT-PCR was performed to measure the expression of oxidative stress and inflammatory response factors in the liver of each group. (H to J) Immunofluorescence staining images and quantification (J) of HO-1 (H) and Nrf2 (I) in the liver. Scale bars, 50 μm.

Based on the aforementioned advantages, we established an ALF model in rats to further evaluate the effectiveness of MCMFs in vivo. The therapeutic transplantation process is depicted in Fig. 5A. A total of 80 ALF rats were divided into 4 groups: normal rats (Control group), sham-operated control group (ALF group), cell transplantation group without the use of microfibers (Cell group), and MCMFs transplantation group (Microfiber group). The number of MSCs, hepatocytes, and HUVECs in both the Cell group and Microfiber group was 10^7^ cells. Liver function indicators, including alanine aminotransferase (ALT) and aspartate aminotransferase (AST), along with survival rate were measured from day 1 to day 7 to assess the degree of liver cell injury (Fig. 5B to D). The results showed that both groups receiving cell transplantation had better outcomes in terms of the aforementioned indicators and survival rate compared to the ALF group. This suggests that the combined transplantation of MSCs, hepatocytes, and HUVECs can effectively control liver failure and treat liver damage. Additionally, the Microfiber group exhibited obviously reduced damage severity and faster recovery compared to the Cell group, indicating a favorable therapeutic effect. To assess the extent of inflammation in the liver, we performed hematoxylin and eosin (H&E) histological staining and Suzuki quantitative scoring on day 3 after transplantation. From the liver tissue sections, it was observed that the necrotic tissue area in the ALF group was larger compared to the liver staining images of the Control group (Fig. 5E and F). However, in both cell-treated groups, the necrotic area markedly decreased, especially in the Microfiber treatment group, where the liver necrosis was minimal, resulting in the lowest Suzuki score. This indicates that the damaged liver was repaired after microfiber treatment.

Due to the key features of inflammatory cytokine storm and excessive oxidative stress in ALF, we further investigated the therapeutic effect of microfiber in reducing inflammation and oxidative stress in vivo. Firstly, we analyzed the expression levels of antioxidant stress genes (*Nfe2l2* and *Ho-1*) in liver tissue through quantitative reverse transcriptase polymerase chain reaction (qRT-PCR) (Fig. 5G). Compared to ALF rats, the expression of these antioxidant stress genes was markedly up-regulated in both groups of rats undergoing cell transplantation, with the Microfiber group showing higher expression than the Cell group. Nrf2 and HO-1 are downstream effectors of ROS signaling with antioxidant functions. HO-1, as a downstream target protein of Nrf2, catalyzes the breakdown of heme into ferrous iron, carbon monoxide, and biliverdin. It can counteract peroxides, peroxynitrites, hydroxyl radicals, and superoxide radicals, making it an important antioxidant enzyme. Immunofluorescence analysis further revealed increased activity of Nrf2 and HO-1 in both cell-transplanted groups, with the Microfiber group exhibiting the highest expression level (Fig. 5H and I). This is consistent with the qRT-PCR results and also aligns with the findings from our previous cell experiments. Additionally, during the progression of ALF, the activation of inflammatory response and the excessive production of inflammatory cytokines accelerate liver damage. To evaluate the inflammatory status of the mouse liver, qRT-PCR was used to analyze the expression of pro-inflammatory cytokines (*Ifng, Tnf, iNos*, and *Il1b*) and anti-inflammatory cytokines (*Cd163, Il10*, and *Il6*) in liver tissue. As expected, the expression of anti-inflammatory cytokines in the liver of the Microfiber treatment group was obviously enhanced, while the expression of pro-inflammatory factors was lower. This indicates better control of the inflammatory response in this group and suggests the important role of implanted Microfiber in liver function recovery.

**Fig. 6.**
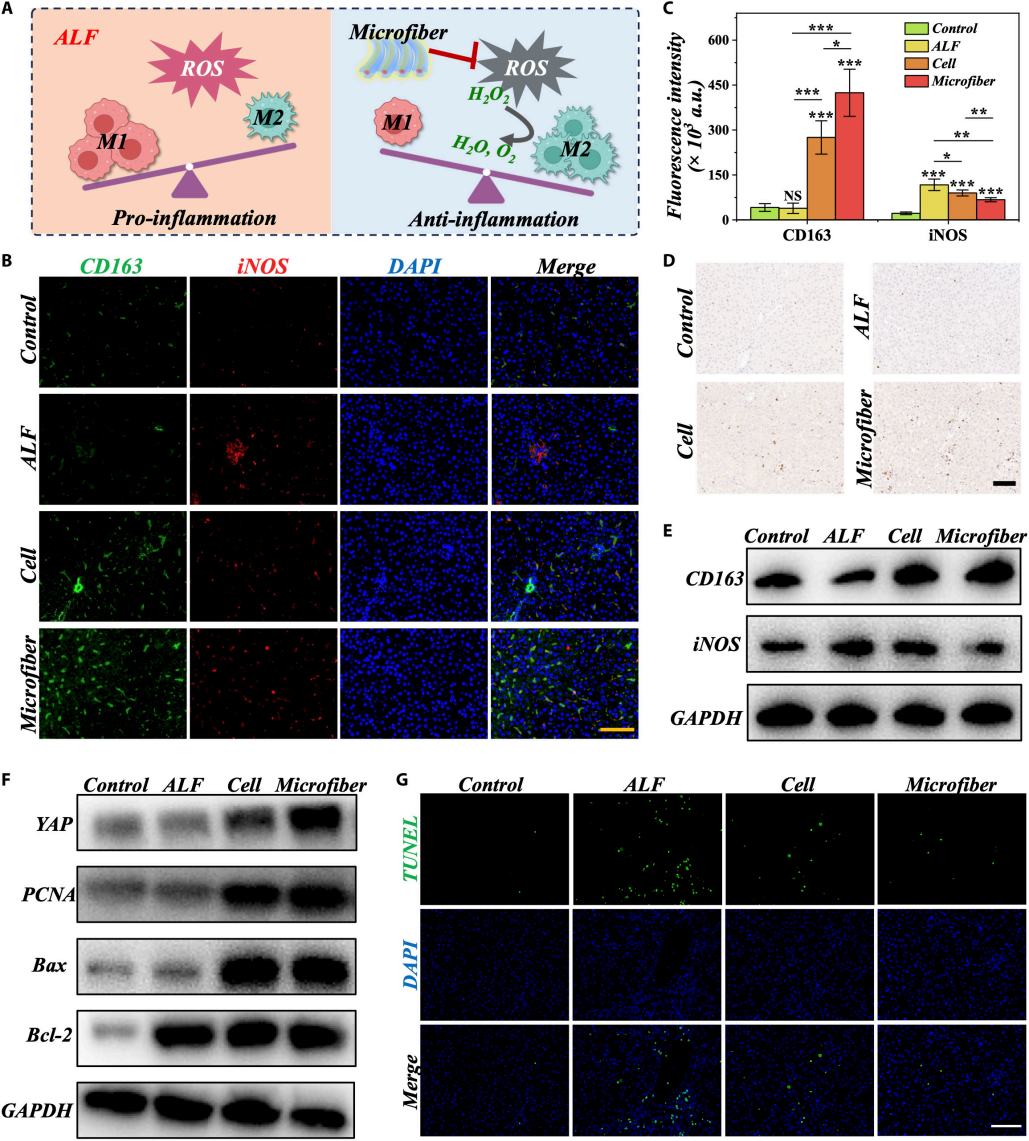
Microfiber promotes liver regeneration and inhibits apoptosis through M2 polarization of macrophages. (A) Schematic representation of macrophage phenotypic reprogramming. (B) Immunofluorescence staining of CD163 (marks M2 macrophages, green) and iNOS (marks M1 macrophages, red) in 4 liver groups, with DAPI staining for cell nuclei (blue). Scale bar, 100 μm. (C) Quantification of fluorescence from (B). (D) Ki67 immunohistochemical staining in the 4 liver groups. Scale bar, 100 μm. (E) Western blotting analysis of CD163 and iNOS expression levels in the 4 liver groups. (F) Western blotting analysis of regeneration and apoptosis in the 4 liver groups. (G) TUNEL immunofluorescence staining in the 4 liver groups. Scale bar, 100 μm.

During ALF, the main mediators of the inflammatory response are macrophages. Liver injury activates macrophages and induces their phenotypic polarization [28,33]. Macrophage polarization refers to the changes in macrophage phenotype in different microenvironments. Macrophages can be divided into 2 distinct phenotypes: M1 and M2, which can convert into each other under different stimuli. M1 macrophages primarily secrete pro-inflammatory cytokines and represent a pro-inflammatory phenotype. On the other hand, M2 macrophages mainly secrete anti-inflammatory factors and promote the repair process, representing an anti-inflammatory phenotype (Fig. 6A). In addition to inhibiting the production of ROS, reducing macrophage inflammatory stress is also crucial in treating ALF. In our previous experiments, we confirmed that microfibers can effectively remove ROS. Thus, we analyzed the expression of polarization-related molecules through immunofluorescence and WB to verify microfibers’ impact on macrophage phenotypic reprogramming. The results showed that, compared to the control group, the ALF group exhibited high expression of the M1 phenotype marker iNOS and low expression of the M2 phenotype marker CD163 (Fig. 6B, C, and E and Fig. S9). However, the Cell group and Microfiber group reversed this M1 phenotype, leading to macrophage polarization toward the M2 phenotype after transplantation therapy. This was primarily reflected in increased CD163 expression and decreased iNOS expression, with the Microfiber group showing more pronounced effects. This indicates that microfibers have an immunomodulatory effect on macrophages, inhibiting M1 polarization and promoting M2 polarization.

Given the important functions of microfibers in ROS clearance and macrophage phenotypic remodeling, we conducted mechanistic studies on their ability to promote liver regeneration and inhibit liver apoptosis. First, we used immunohistochemistry to assess cell proliferation by staining liver tissue sections with Ki-67. It was evident that liver regeneration was suppressed in the ALF group (Fig. 6D and Fig. S10A). Additionally, compared to the other 3 groups, the Microfiber group showed improvement in liver cell proliferation. This result was further validated by Western blot analysis of liver tissue, which showed decreased expression of Yes-associated protein 1 (YAP) and proliferating cell nuclear antigen (PCNA) in the ALF group, while the Microfiber group rescued liver regeneration inhibition and promoted recovery (Fig. 6F and Fig. S11). Furthermore, we performed terminal deoxynucleotidyl transferase-mediated dUTP nick-end labeling (TUNEL) immunofluorescence staining on liver tissue to assess apoptosis levels. In contrast to the regenerative outcomes, the ALF mice exhibited increased apoptotic cells in the liver, whereas the Microfiber group showed a reduction in apoptosis levels (Fig. 6G and Fig. S10B). Moreover, the expression of the apoptosis-inhibiting protein B-cell lymphoma-2 (Bcl2) was increased in the Microfiber group, while the expression of the apoptosis-promoting protein Bcl2-associated X (Bax) was suppressed. This indicates that the Microfiber group has a regenerative and anti-apoptotic effect on cells in the ALF.

## Conclusion

In conclusion, inspired by the natural structure of liver microunits, we have designed a coaxial multichannel microfluidic chip that can continuously produce lobules with a multicellular spatial distribution. Compared to previous studies using patterned multiprint head systems [34], our method offers several unique advantages, including continuous production and scalability, high spatial precision, and the use of biodegradable and biocompatible hydrogels to enhance therapeutic outcomes. Most importantly, the alternation of hepatocytes and MSCs around HUVECs is a key feature of our design, which enhances cell functionality and promotes liver regeneration. This innovative arrangement not only provides a biomimetic microenvironment that supports cell survival and function but also facilitates intercellular interactions, which are crucial for liver repair and regeneration.

Firstly, the cascading inflammatory response is a key factor in ALF [28]. MSCs can secrete various cytokines, such as PGE2, IL-4, and IL-10, to modulate the inflammatory environment and support hepatocyte function [35,36]. Moreover, MSCs can influence macrophage phenotypic polarization and specific cell death pathways in hepatocytes, such as pyroptosis and necroptosis, thereby reducing liver injury and promoting liver regeneration through pathways like Wnt and YAP [37–40]. This spatial arrangement ensures that these factors are effectively delivered to hepatocytes, maximizing their therapeutic potential. Another key mechanism in ALF is the production of ROS, which can cause oxidative stress and liver damage. The Nrf2/HO-1 pathway, activated in our MCMFs, is a critical defense mechanism against oxidative stress, with Nrf2 regulating the expression of antioxidant enzymes such as HO-1. In our biomimetic lobules, MSCs and HUVECs activate this pathway, helping to reduce oxidative damage and promote liver regeneration.

Secondly, in addition to paracrine effects, the spatial arrangement of cells in MCMFs also promotes direct cell–cell interactions. Hepatocytes and MSCs can form junctions with HUVECs, facilitating the exchange of signaling molecules. These direct interactions are crucial for coordinating cellular responses during liver regeneration and repair. Moreover, the alternation of hepatocytes and MSCs around HUVECs promotes the formation of functional sinusoidal structures. In the liver, sinusoids are lined by endothelial cells and interspersed with hepatocytes, facilitating the exchange of nutrients, oxygen, and metabolic products. MCMFs could replicate this structure, enabling efficient nutrient delivery and waste removal, which are essential for maintaining hepatocyte viability and function.

In summary, the alternation of hepatocytes and MSCs around HUVECs in MCMFs enhances cell functionality. This spatial arrangement supports paracrine signaling, promotes the formation of functional structures, modulates inflammation, and facilitates direct cell–cell interactions. These features endow our MCMFs with the ability to reduce oxidative stress, reprogram macrophages, and optimize the microenvironment for ALF treatment, thereby promoting the repair of damaged livers. Therefore, MCMFs offer distinct advantages in treating related diseases and enhancing traditional clinical methods. Given that the in vivo degradation rate of Alg is influenced by its concentration, different cross-linking agents, and pH, future research will focus on elucidating the in vivo degradation kinetics of Alg to align it with the rate of liver regeneration [41,42]. Future studies should also further explore the detailed molecular mechanisms underlying these effects and investigate the long-term therapeutic potential of MCMFs in chronic liver diseases.

## Methods

### Fabrication of the coflow microfluidic chip platform

There are 7 parallel capillary glass tubes with an inner diameter of 500 μm. They are arranged in a 2-3-2 configuration from top to bottom. The 6 tubes surrounding the outer side are collectively referred to as Tube I, while the single capillary glass tube in the middle is referred to as Tube II. Another capillary glass tube with an inner diameter of 120 μm (Tube III) is inserted into the middle of Tube II. Then, Tubes I to III are inserted into a capillary glass tube with a conical outlet and an inner diameter of 2.4 mm, and an outlet diameter of 1 mm (referred to as Tube IV). A circular tube (referred to as Tube V) is fixed in the appropriate position on the glass slide, leaving enough space for the other tubes on the slide. Tubes I to IV are inserted in reverse through Tube V using a capillary tube with an inner diameter of 1.5 mm (referred to as Tube VI), ensuring that the tip of Tube IV is inserted into Tube V without completely blocking its opening, and both tubes are secured. Finally, 6 tapered and curved capillary vessels are inserted into Tube I, and needle tips are fixed at the connection points of all the tubes to facilitate liquid input.

### Fabrication of microfibers

To obtain the solid 6-component microfibers, a 1.0 wt.% Na-Alg solution dyed with green and blue fluorescent dyes is alternately introduced into the 6 channels (Tube I) of the chip device through the middle channel. Simultaneously, a 2.0 wt.% CaCl_2_ fluid is introduced into the device through the outer channels (gaps between Tubes IV and VI). By closing off the other channels, solid 6-component microfibers can be generated in situ. To obtain solid microfibers with a total of 7 components, an additional step can be taken. By opening the channel between Tube II and Tube III and introducing a Na-Alg pre-gel solution dyed with red fluorescent dye, solid microfibers with a total of 7 components can be obtained. Furthermore, if a 10.0 wt.% PVA solution mixed with 1.0 wt.% CaCl_2_ is introduced into Tube II as the inner phase, hollow microfibers with a total of 7 components can be generated. Syringe pumps (Harvard PHD 2000 series) were used to control and regulate the flow rates of various solution.

### Fabrication of MCMFs

The method of MCMFs is consistent with the preparation of hollow microfibers with a total of 7 components. In this process, HUVECs (2.5 × 10^5^ cells/ml) were added to the gaps between Tubes II and III to prepare the Na-Alg solution. In Tube I, the 6 channels were sequentially filled with MSCs (2.5 × 10^5^ cells/ml) and hepatocytes (5 × 10^5^ cells/ml) to prepare the Na-Alg solution. The fabricated MCMFs were then cultured for future use. MSCs, HUVECs, and human induced pluripotent stem cells (hiPSCs) were sourced from the Clinical Stem Cell Center at Nanjing Drum Tower Hospital, while hepatocytes were induced from hiPSCs following our previously established method (*12*).

### In vitro function

Four groups were studied in this experiment: 2D plate (control group); hollow microfibers loading hepatocytes (hepatocyte microfiber group); hollow microfibers loading hepatocytes, MSCs, and HUVECs (multicell microfiber group); and MCMFs. Each group consisted of 10^6^ hepatocytes. ALB and urea levels were measured using ELISA, while CYP levels were assessed using qRT-PCR.

### ROS-scavenging assessment

To simulate acute liver injury in vitro, LPS (100 ng/ml) was used to stimulate the 4 groups: 2D plate (control group); hollow microfibers loading hepatocytes (hepatocyte microfiber group); hollow microfibers loading hepatocytes, MSCs, and HUVECs (multicell microfiber group); and MCMFs. Each group contained 10^6^ hepatocytes. Subsequently, cells were incubated with DCFH-DA (10 μM, Sigma-Aldrich) for 30 min and observed under a confocal microscope to measure ROS levels.

### Animal experiments

A total of 80 rats with ALF were divided into 4 groups: normal rats (Control group), sham-operated control group (ALF group), cell transplantation group without microfibers (Cell group), and MCMFs transplantation group (Microfiber group). The induction of ALF in rats was achieved by an intraperitoneal injection of D-galactosamine hydrochloride (D-Gal) at 0.6 g/kg for the D-Gal-induced model. The Cell group and Microfiber group both received 10^7^ MSCs, hepatocytes, and HUVECs. All rats survived after the laparotomy procedure. Over the next 7 days, the survival rates were recorded daily, and the levels of ALT and AST were measured. The livers of the rats from the different groups were collected for further mechanistic studies. This study strictly followed the guidelines set by the Animal Ethics Committee of the Drum Tower Hospital, affiliated with the Medical School of Nanjing University (No. 20230401).

### Histological analysis

Liver tissue fixed in 4% paraformaldehyde was embedded in paraffin, sectioned, and stained for antibody. The stained sections were then observed under a microscope for histological analysis.

### qRT-PCR assay

Total RNA was extracted from cells or liver tissue using Trizol reagent (Thermo Fisher Scientific). The purified RNA was reverse-transcribed into cDNA using the Prime Script Reverse Transcription Kit (Takara Bio, Shiga, Japan) and then subjected to PCR using SYBR Premix Ex Taq (Takara Bio) on the ABI7500 detection system.

### Western blotting analysis

Proteins were extracted from liver tissue or cells using radioimmunoprecipitation assay buffer (KeyGen Biotech, Nanjing, China) supplemented with phenylmethylsulfonyl fluoride at 4°C. The protein concentration was determined using the BCA Protein Assay Kit (KeyGen Biotech, Nanjing, China). Protein immunoblotting was performed following established experimental protocols, with all protein antibodies purchased from Abcam Cambridge, MA, USA.

### Immunofluorescence staining

Frozen sections were fixed using a 4% formalin solution, washed 3 times, and blocked before overnight incubation with the primary antibody. Following 3 additional washes, the sections were incubated with a secondary antibody containing a fluorescent group. After 3 more washes, the nuclei were stained, and the sections were observed using a fluorescence microscope before sealing. All images presented in the results are representative of at least 3 images.

## Data Availability

The data that support the findings of this study are available from the corresponding authors upon reasonable request.
