# Supplementary material for: Bioinspired Spatially Ordered Multicellular Lobules for Liver Regeneration
Source: Research (Wash D C). 2025 Mar 17;8:0634. doi: 10.34133/research.0634 (PMC11912749; doi:10.34133/research.0634)
Supplement: Supplementary 1 — Supporting information [file research.0634.f1.pdf]

Bioinspired spatially ordered multicellular lobules for liver regeneration

Jinglin Wang, Danqing Huang, Haozhen Ren*, Yuanjin Zhao*

Additional information

Supplementary Information accompanies this paper.

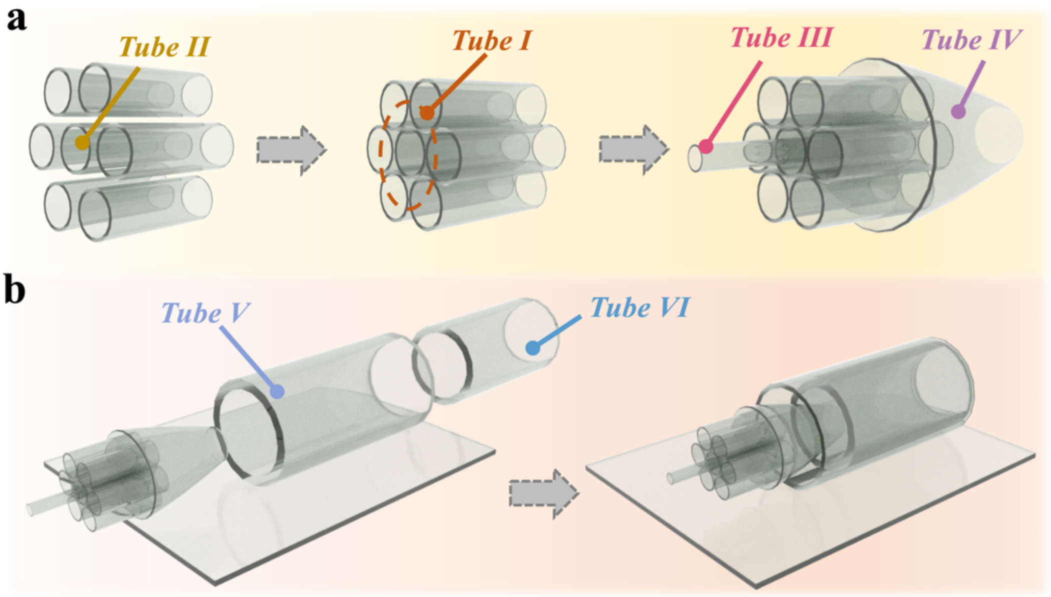

Figure S1. Construction of the microfluidic chip platform. (a) Seven parallel capillary glass tubes assembled coaxially (Tubes I, II) with a capillary glass tube (Tube III) inserted in the center, then inserted into a tapered outlet capillary glass tube (Tubes IV). (b) Stacking of Tubes I to VI and fixed onto a glass slide.

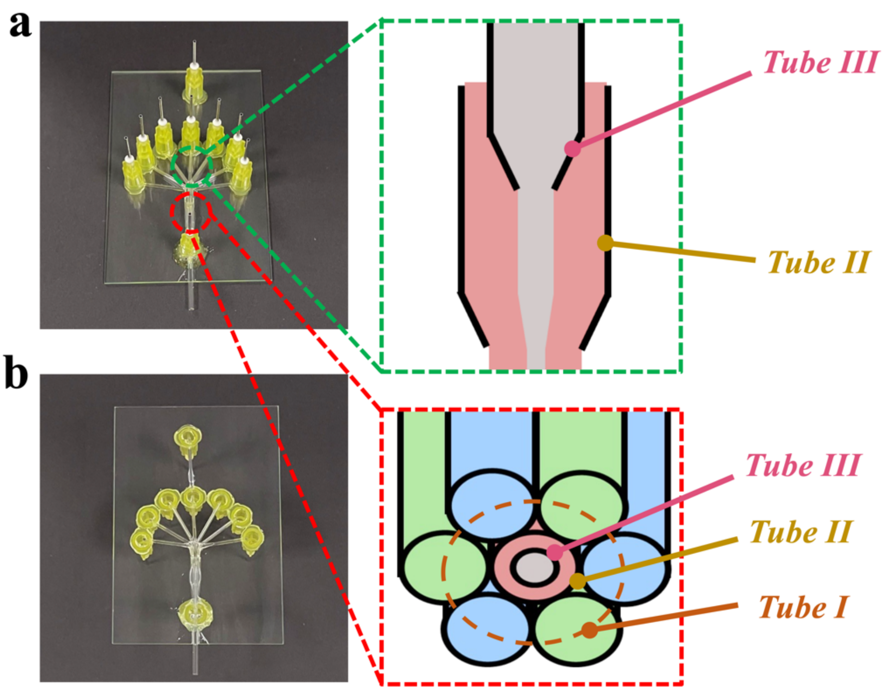

Figure S2. Front (a) and back (b) photographs of the microfluidic chip platform.

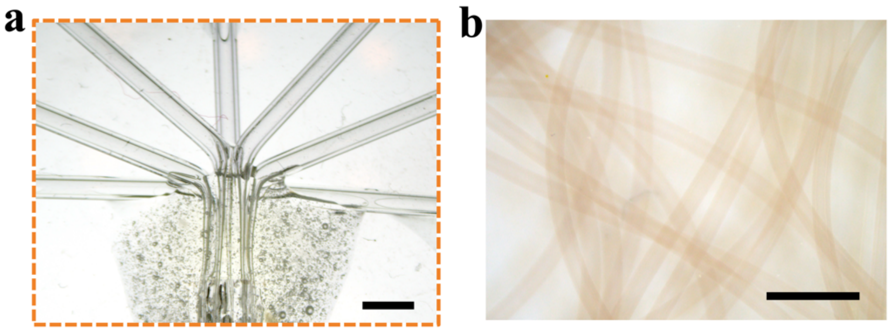

Figure S3. (a)Stereomicroscopic image of the joints in the assembly of parallel capillary glasses. Scale bar is 1mm. (b) The optical micrograph of hollow microfibers. Scale bar is 1 mm.

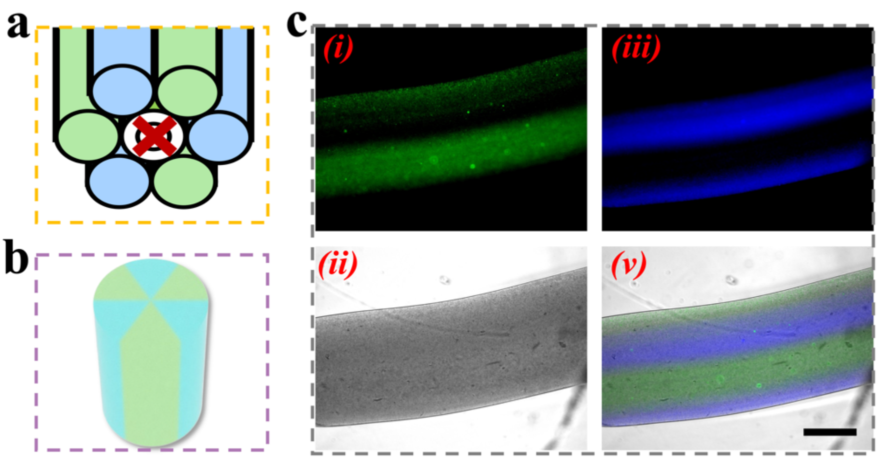

Figure S4. The solid multi-component microfiber formed by the 6 channels in Tube I. (a) Schematic representation of the distribution of green and blue fluorescent liquids inside Tube I. (b) Schematic representation of the microfiber generated from Tube I. (c) Fluorescence imaging of the multi-component fibers formed by the 6 channels: (i) Green fluorescence channel, (ii) Bright field channel, (iii) Blue fluorescence channel, (iv) Merged image after combining the channels. Scale bar is 500 μm.

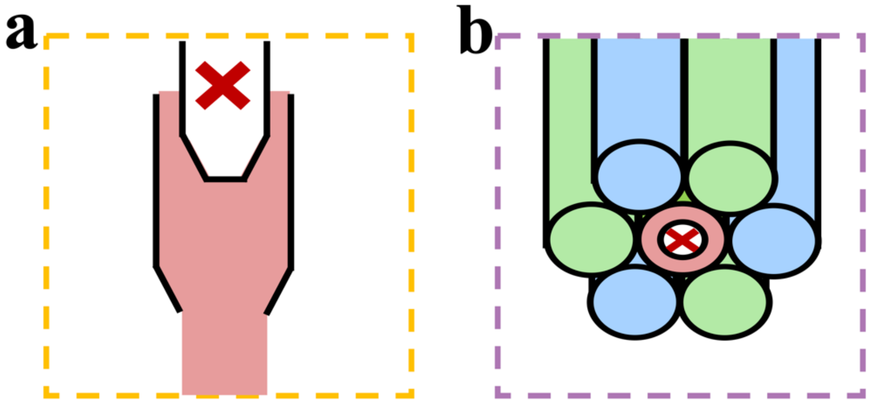

Figure S5. The multi-component microfiber formed by the 7 channels in Tube I and gaps between Tube II and III. (a) Schematic representation of the distribution of red fluorescent liquids inside the gaps between Tube II and III. (b) Schematic representation of the distribution of red, green and blue fluorescent Na-Alg solutions inside Tube I and gaps between Tube II and III.

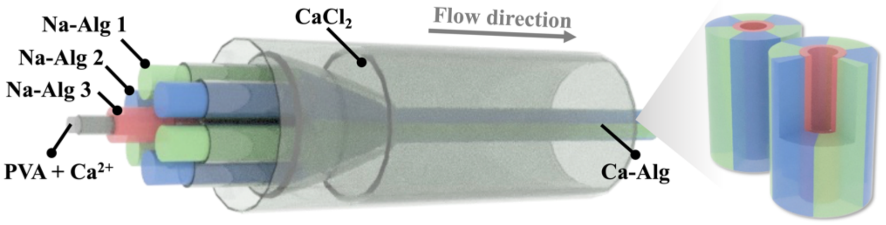

Figure S6. Schematic illustration of the microfluidics technology utilized in the coaxial chip platform to create hollow vertical multi-compartmental microfibers that emulate liver lobules.

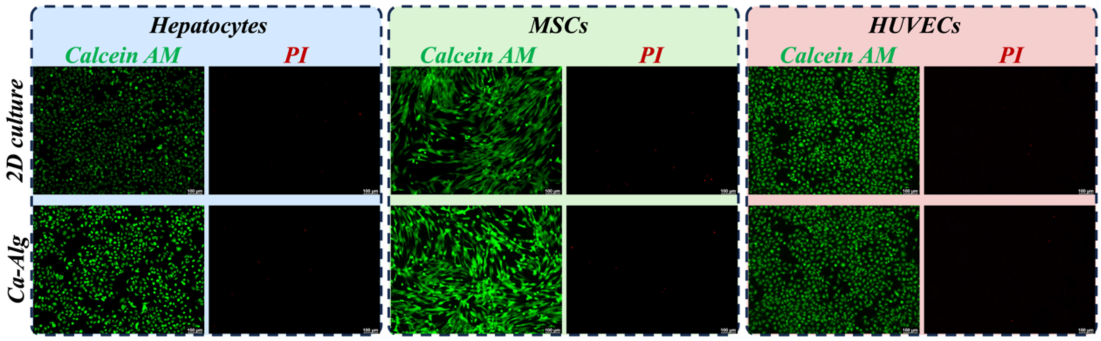

Figure S7. Live-dead staining was performed on hepatocytes, MSCs, and HUVECs in 2D culture and Ca-Alg group. Calcein AM was used to stain live cells, which emits green fluorescence. PI was used to stain dead cells, which emits red fluorescence. The scale bar is 100 μm.

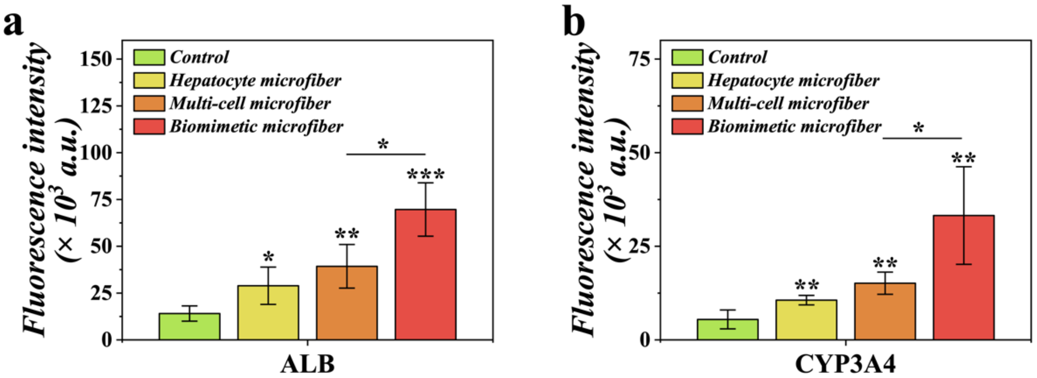

Figure S8. The fluorescence quantification levels of ALB (a) and CYP3A4 (b).

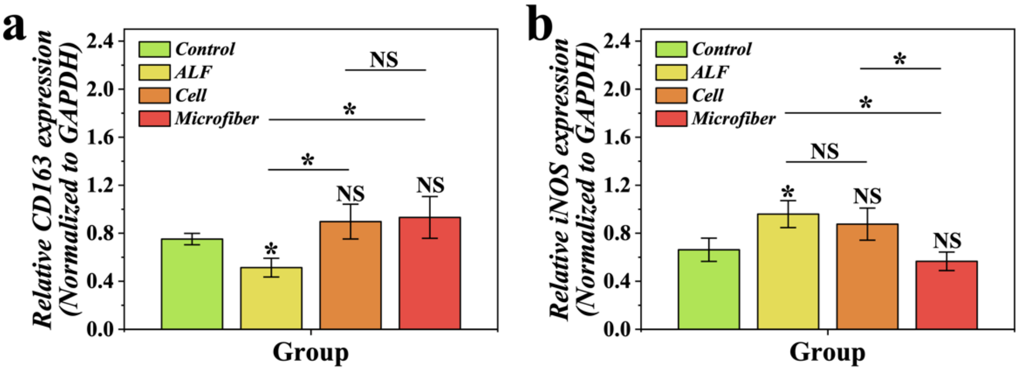

Figure S9. The quantitative levels of CD163 (a) and iNOS (b) in the four liver groups are assessed using Western blotting.

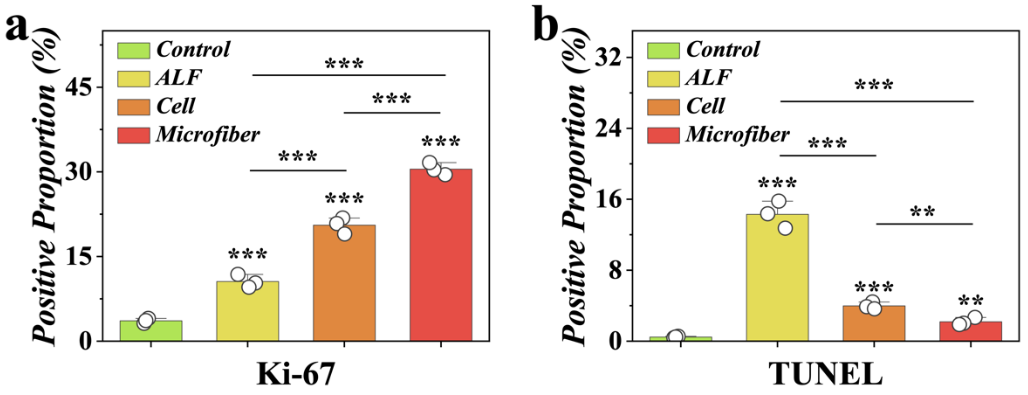

Figure S10. The levels of Ki67 (a) and TUNEL (b) staining were quantified in the four liver groups.

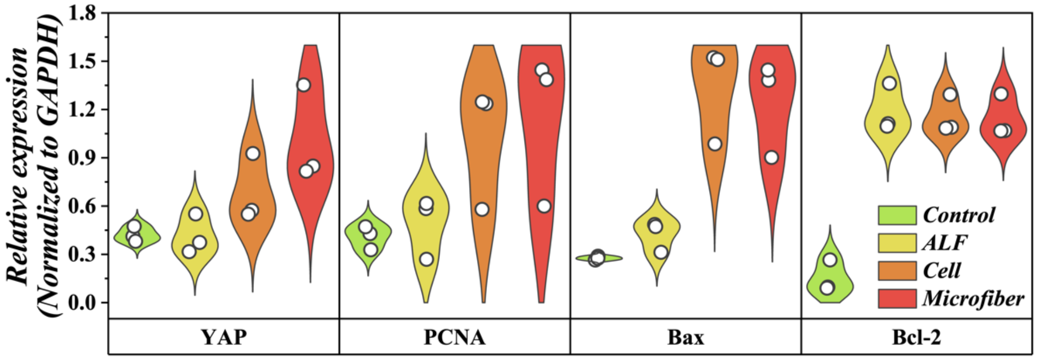

Figure S11. The quantitative levels of regeneration and apoptosis in the four liver groups are assessed using Western blotting.

Table 1. Primers for qRT-PCR.

Gene		Primer
Ifng	F	GCTGATGGCCTGGTTGTCTTTC
	R	CGCCGCGTCTTGGTTTTG
Nfe2l2	F	TCCTCTGCTGCCATTAGTCA
	R	GTGCCTTCAGTGTGCTTCTG
Tnf	F	ATGGGCTCCCTCTCATCAGT
	R	AAATGGCAAATCGGCTGACG
iNos	F	GGAGAAAACCCCAGGTGCTA
	R	GTGGTGAAGGGTGTCGTGAA
Ho-1	F	CTTTCAGAAGGGTCAGGTGTC
	R	TGCTTGTTTCGCTCTATCTCC
Il1b	F	GCCTCAAGGGGAAGAATCTATACC
	R	GGGAACTGTGCAGACTCAAACT
Cd163	F	TGTAGTTCATCATCTTCGGTCC
	R	CACCTACCAAGCGGAGTTGAC
Il10	F	CTGGCTCAGCACTGCTATGT
	R	GCAGTTATTGTCACCCCGGA
Il6	F	CCTACCCCAACTTCCAATGCT
	R	GGTCTTGGTCCTTAGCCACT
GAPDH	F	GAGTCAACGGATTTGGTCGT
	R	TTGATTTTGGAGGGATCTCG

Table 2. Antibodies for immunofluorescence and Western blotting.

Antibody	Cat. No.	Company
HO-1	ab189491	Abcam
Nrf2	ab62352	Abcam
ALB	ab207327	Abcam
CYP3A4	ab245774	Abcam
CD163	ab182422	Abcam
iNOS	ab178945	Abcam
YAP	ab76252	Abcam
PCNA	ab29	Abcam

Bax	ab32503	Abcam
Bcl-2	ab194583	Abcam
GAPDH	ab8245	Abcam
